# Supplementary material for: Formulation of SARS-CoV-2 Spike Protein with CpG Oligodeoxynucleotides and Squalene Nanoparticles Modulates Immunological Aspects Following Intranasal Delivery
Source: Pharmaceutics. 2022 Nov 21;14(11):2539. doi: 10.3390/pharmaceutics14112539 (PMC9693849; doi:10.3390/pharmaceutics14112539)
Supplement: Supplementary file 1 [file pharmaceutics-14-02539-s001.zip › pharmaceutics-2027005-supplementary.pdf]

pharmaceutics

MDPI

Supplementary Materials

for

Formulation of SARS-CoV-2 Spike Protein with CpG Oligode-oxynucleotides and Squalene Nanoparticles Modulates Immunological Aspects Following Intranasal Delivery

Hui-Min Ho, Chiung-Yi Huang, Chung-Hsiang Yang, Shih-Jen Liu, Hsin-Wei Chen, Guann-Yi Yu, Jen-Kun Chen, Tsung-Hsien Chuang and Ming-Hsi Huang *

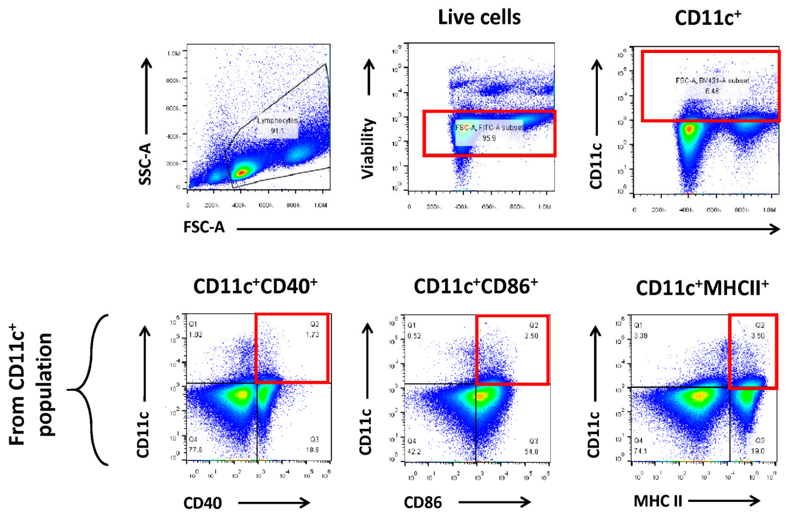

Figure S1. Gating strategy of the FACS experiment on surface expression of APCs at superficial cervical draining lymph nodes (LNs) following intranasal vaccination with PELC:CpG-formulated S protein. Dead cells were excluded from analysis by staining with Live/Dead™ cell viability assay kit. The cells were further gated for the levels of CD11c^{+}, CD11c^{+}CD40^{+}, CD11c^{+}CD86^{+}, or CD11c^{+}MHCII^{+} cells.

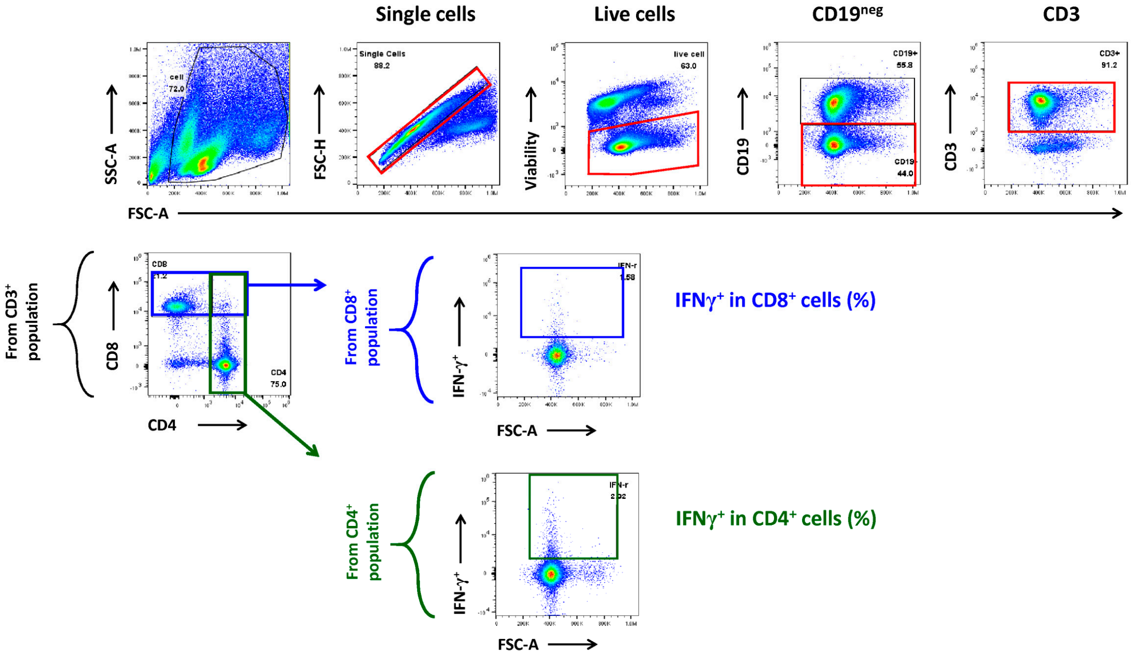

Figure S2. Gating strategy of the FACS experiment on IFN-γ-secreting cells in antigen-specific $CD4^+$/$CD8^+$ T cells following intranasal vaccination with PELC:CpG-formulated S protein. Splenic $CD19^{neg}CD3^+$ cells were further gated for the levels of IFN-γ expression in $CD4^+$ or $CD8^+$ cells.
